# Volatiles Accumulation during Young Pomelo (*Citrus maxima* (Burm.) Merr.) Fruits Development

**DOI:** 10.3390/ijms23105665

**Published:** 2022-05-18

**Authors:** Nan Xiang, Yihan Zhao, Bing Zhang, Qiuming Gu, Weiling Chen, Xinbo Guo

**Affiliations:** 1 Guangdong Province Key Laboratory for Green Processing of Natural Products and Product Safety, School of Food Science and Engineering, Engineering Research Center of Starch and Vegetable Protein Processing Ministry of Education, South China University of Technology, Guangzhou 510640, China; nanxiang0908@163.com (N.X.); zhaoyihan19980515@163.com (Y.Z.); dbkxydd@163.com (B.Z.); 2 Southern Golden Pomelo Research Institute of Meizhou, Meizhou 514743, China; guqm@163.com (Q.G.); chenweiling1011@163.com (W.C.)

**Keywords:** volatiles, young pomelos, fruit ripening, transcriptomic, transcription factors

## Abstract

As widely planted fruits with high nutritional and medical values, pomelos are managed systematically to achieve the largest economic benefits. But the annual shedding of young pomelos, which could be applied as feedstocks for essential oil extraction with their abundant volatiles, leads to a waste of source. The present study selected two commonly planted pomelo (*Citrus maxima* (Burm.) Merr.) varieties in Southern China, to investigate the volatile profiles during young pomelo fruits development. Combing transcriptomic analysis, this study aimed at identifying the prominent volatile components in young pomelo fruits in order to preferably extract profitable volatiles, as well, increasing the knowledge concerning regulatory roles of transcription factors (TFs) on volatiles accumulation in young pomelos. Totally 29 volatiles were identified, including 14 monoterpenoids and 13 sesquiterpenoids. Diprene was the principal component with the highest amount. Volatiles were generally decreased during fruits development but preferable stages were figured out for volatile collections. 12 and 17 TFs were related to developing time while *ERF003* and *MYC2* were highly correlated to monoterpenoids. These findings put forward the comprehensive usages of young pomelos and enriched the regulatory roles of TFs on both fruit development and volatiles metabolism.

## 1. Introduction

Pomelos (*Citrus maxima* (Burm.) Merr.) are widely planted around the world and possess high nutritional and medical values, owing to their abundant phytochemicals content as well as superior antioxidant and anti-inflammatory activities [1,2,3]. China is one of the leading producers of pomelo crops worldwide [4], where pomelos are not only consumed as fruits, but also applied in folk medicine [3]. Pomelos peels have been processed into plenty of byproducts, including juice, tea, and jams [5]. In order to release space and avoid nutrient competition for pomelo fruits development, the shedding of young pomelos is often operated artificially as the management of pomelo trees. Hence the annual shedding of enormous young pomelos causes a huge waste of sources, as the output of young pomelos even exceeds ripe pomelo fruits. Under these circumstances, young pomelo fruits are collected as materials for extracting naringin, the natural food additives. However, the extraction still counts against the comprehensive utilization of the pomelo source, since volatiles are easily lost during processing.

Volatiles are ubiquitously accumulated in special plant cells and structures, from where they could be released. They have received much attention in terms of their biological functions on plants environmental adaptation and survival, as well as their applications in industries, such as food additives for specific aroma, materials for industrial pharmaceuticals and biofuels [6]. Essential oils, as the most vital by-products of citrus processing, are widely employed in air-fresheners, household cleaning products, perfumes, cosmetics, and medicines [7]. The volatiles composition in peels of several pomelo varieties have been studied, with terpenoids occupying a large proportion, including β-pinene, α-pinene, β-myrcene, germacrene D, linalool, cis-linalool oxide, sabinene, and nootkatone [5]. Hence, pomelo peels are often applied as sources for essential oil extraction [5]. In addition, hydrocarbons such as limonene, pinene, terpinene and copaene as well as terpenoids linalool, neorl and geraniol provided citrus-based flavors in pomelo juice [8]. During the ripening of pomelo fruits, limonene, terpinene, pinene, and citral contents were highest in matured fruits, whereas sesquiterpenes were reported with declinations during developmental process [8]. As for unripe fruits, the essential oil yielded from unripe *Citrus limon* fruits was impressive as compared to ripe fruits, which comprised abundant volatile components [9]. Therefore, young pomelo fruits are supposed to be alternative sources for pomelo essential oil extraction. For the sake of making full use of young pomelo fruits, the investigation on volatiles during development, which could provide guidance for selecting best shedding time for young pomelo fruits with abundant specific volatiles, is necessary.

Besides, the majority volatiles in pomelos were classified into terpenoids, which are generated from mevalonate and methylerythritol phosphate pathways. The candidate genes in regulating carotenoid biosynthesis in pomelo cultivars with different colors have been reported [10], hence indicating the possible regulatory roles of transcription factors on terpenoids biosynthesis. In Southern China, two varieties of pomelos, Golden Pomelo (*Citrus maxima* (Burm.) Merr. cv. *Jinyou*, GP) and Honey Pomelo (*Citrus grandis* cv. *Miyou*, HP), are widely planted and predominantly traded [11]. Similarity in outlooks, the two varieties are discrepant in flavors. They were selected in the present study for investigating dynamic changes in volatiles during the development of their young fruits. Transcriptomic analysis was also combined to distinguish the key transcription factors that participated in modulating volatiles accumulation. The present work could reveal volatiles composition and variation in young pomelo fruits during development, besides, provide guidance for shedding and facilitate the application of young pomelo fruits as alternative sources for essential oil extraction, what’s more, broaden the knowledge on regulatory mechanism of principal volatiles in pomelos.

## 2. Results

### 2.1. RNA Sequencing Results and Time Cluster Patterns

**Figure 1:**
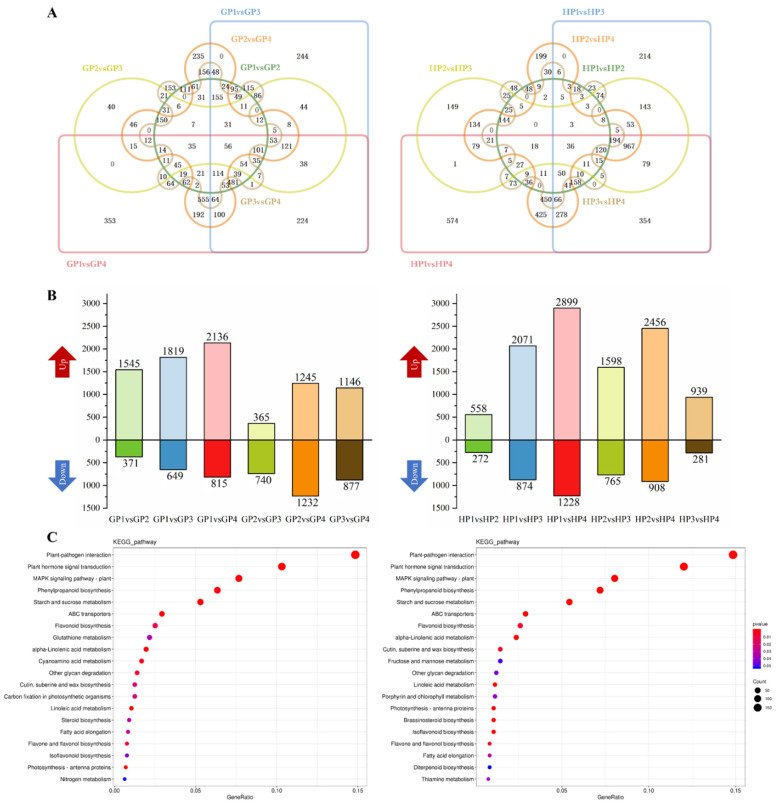
The transcriptomic results of young pomelo fruits. (**A**) Venn plot of six comparing groups in two varieties of young pomelo fruits. (**B**) The up- and down-regulated DEGs in two varieties of young pomelo fruits (FC > 2, *p* < 0.01). (**C**) KEGG enrichments of DEGs in two varieties of young pomelo fruits.

According to transcriptomic results, the differently expressed genes (DEGs) in each variety between two developmental stages were filtered by Fold Change (FC) > 2, *p* < 0.01. Venn plots were depicted in each variety among the six comparing groups comprising four stages (Figure 1A). Besides, the apparently up- and down-regulated DEGs were distinguished in Figure 1B. Fruits ripening obviously resulted in the upregulation of a large amount of DEGs as the latter stage generally held more abundant up-regulated DEGs than the previous stage. Comparatively, the increased effects on DEGs from fruit ripening were obvious in HP variety. KEGG enrichments were operated on the up-regulated DEGs in GP and HP, respectively (Figure 1C). The most abundant seven pathways were similarly enriched in both GP and HP, involving in plant hormones, phenylpropanoids, flavonoids, starch and sucrose. Besides, alpha-linolenic acid, linoleic acid metabolism, fatty acid elongation and diterpenoid biosynthesis that were related to volatiles formation were also identified.

**Figure 2:**
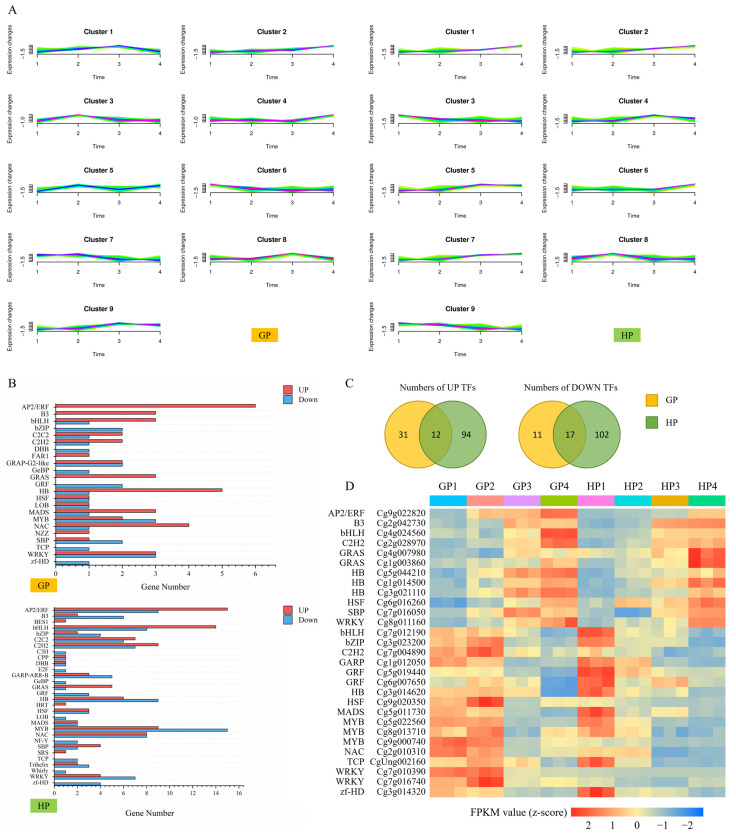
Screening of transcription factors (TFs). (**A**) Time cluster results in two varieties of young pomelo fruits. (**B**) TFs enrichments in specific clusters. In GP, clusters 2 and 7 were separately selected for enriching up- and down-regulated TFs during fruits development; In HP, clusters 1, 2 and 7were selected for enriching up-regulated TFs while clusters 3 and 9 were selected for enriching down-regulated TFs during fruits development. (**C**) Venn plots of the up- and down-regulated TFs in GP and HP varieties. (**D**) The FPKM values of TFs that belonged to the intersection of GP and HP varieties.

Cluster analysis was separately performed on DEGs in GP and HP (Figure 2A, Table S1). The cluster groups were set as nine in order to distinguish the discrepant changing patterns of DEGs. DEGs that were clustered in Cluster 2 in GP and in Clusters 1, 2 and 7 in HP separately represented the kept increasing DEGs. Correspondingly, the expressions of DEGs in Cluster 7 in GP and in Clusters 3 and 9 in HP were decreased during fruit development. On the basis of cluster results, transcription factors (TFs) were predicted, respectively in up- and down-regulated DEGs in both GP and HP varieties (Figure 2B). Totally, 23 and 30 families were involved, among which, AP2/ERF family comprised the largest proportion, while the number of the downregulated TFs was the highest in MYB family in both GP and HP varieties. Venn graphs showed 12 consistently up-regulated and 17 down-regulated TFs in both GP and HP varieties (Figure 2C). The FPKM values were standardized and depicted in Figure 2D. Generally, 12 TFs were from 9 families, whose expressions kept increasing during fruit development. Conversely, the expressions of 17 TFs classified in 13 families were reduced. Summarily, the key TFs that would participate in fruit development and metabolites accumulation were identified in young pomelo fruits.

### 2.2. Volatiles Components in Young Pomelo Fruits

**Figure 3:**
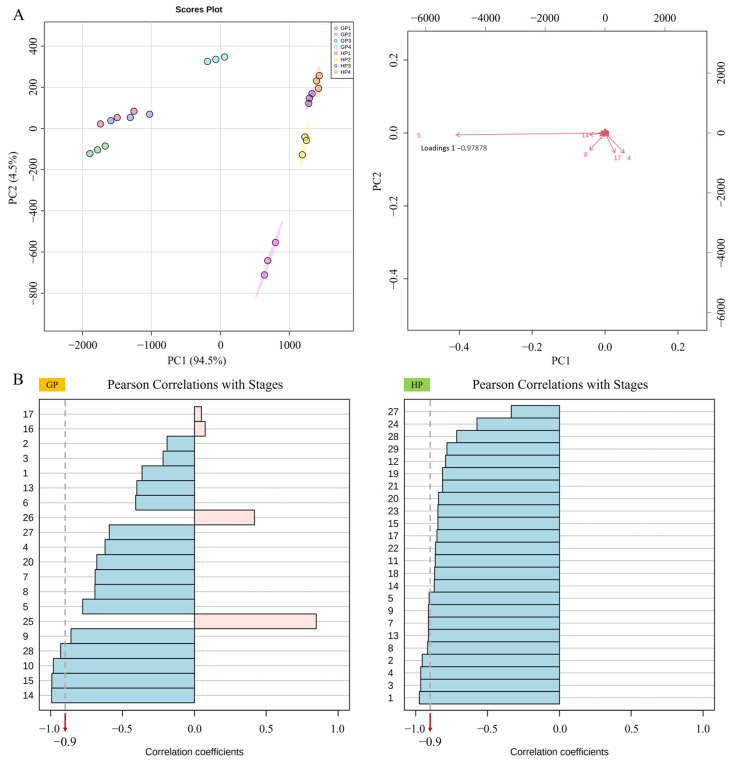
Volatile profiles in young pomelo fruits. The numbers stand for volatiles which could be referred in Table S2. (**A**) PCA analysis results. (**B**) The coefficients of volatiles correlated to development stages in two varieties of young pomelo fruits.

GC-MS/MS identified 29 volatile compounds in the present study (Table S2). Principal component analysis was conducted and results were shown in Figure 3A. PC1 and PC2 separately explained 94.5% and 4.5% of the variation, totally 99.0%. As the loadings results of volatiles shown, No. 5 volatile, diprene, held the negative correlation value with PC1 as −0.979, indicating the most negative relationship to PC1. Diprene was the most abundant volatiles in young pomelo fruits, as it comprised around 70–91% and 33–45% of the total volatile content in GP and HP, respectively. At stage 1, in GP, caryophyllene (around 11%) and beta-ocimene (around 8%) were the richest components following diprene, diversly, it was beta-myrcene (20–30%) and germanene D (10–20%) in HP. Figure 3B depicted the relationship between volatiles and time patterns, presented by coefficient values. The volatiles that varied negatively to developmental stages comprised a large proportion of the total. No. 9, 14 and 15 components separately referred to cosmene, caryophyllene and humulene which exhibited negative correlations with developing time in GP and HP varieties. Taking coefficient value −0.9 as the dividing benchmark, the number of volatiles that negatively correlated to the increasing development time in HP was larger than that in GP. Apart from the above-mentioned volatiles, trans-limonene oxide and hexadecanoic acid, methyl ester were prominently decreased in GP, while orthodene, beta-pinene, beta-phellandrene, beta-myrcene, diprene, trans-beta-ocimene, beta-ocimene, cosmene and linalool were distinguished in HP.

**Figure 4:**
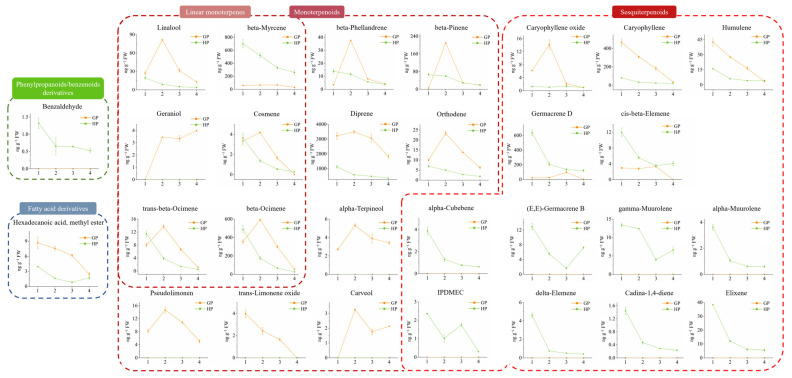
Varied contents of volatiles during young pomelo fruits development. IPDMEC: (1R,7S,E)-7-Isopropyl-4,10-dimethylenecyclodec-5-enol.

Volatiles were classified into four commonly reported volatile categories, including phenylpropanoids/benzenoids derivatives, fatty acid derivatives, monoterpenoids and sesquiterpenoids (Figure 4). Except for the benzaldehyde and hexadecenoic acid (HDA), methyl ester that separately generated from the degradations of benzenoids and fatty acids, majority of volatiles were classified into monoterpenoids and sesquiterpenoids. Benzaldehyde was uniquely detected in HP with its decrease pattern during young pomelos development, while hexadecenoic acid, methyl ester was abundant in GP but reduced to the same level at stage 4 as in HP.

Totally 14 monoterpenoids were detected, 6 of which were linear monoterpenoids. 5 monoterpenoids were uniquely existed in GP, where other monoterpenoids were highly accumulated as compared to HP. Distinctly, GP at stage 2 increased contents of beta-pinene, beta-phellandrene, orthodene, diprene, cosmene, trans-beta-ocimene, beta-ocimene, alpha-terpineol, pseudolimonen, carveol, and linalool to the highest levels. However, those volatiles were decreased in the following developing stages. Dramatically, trans-limonene oxide gradually reduced to zero during fruit development while geraniol accumulated from zero at the initial to the maximum level at stage 4 in GP. The eight identified monoterpenoids in HP consistently decreased during fruits development.

As mentioned to sesquiterpenoids, 13 components were identified in HP, but only 5 of them were detected in GP. GP possessed more caryophyllene oxide, caryophyllene and humulene contents than HP, but the contents gradually decreased to similar levels as compared to HP at stage 4. In general, sesquiterpenoids in HP reduced from stage 1 to 2 sharply and kept decreasing during the following developing stages, whereas (E,E)-germacrene B and gamma-muurolene were slightly increased from stage 3 to 4. In summary, majority of the detected volatiles were terpenoids, whose concentrations reduced during young pomelo fruits development. GP contained abundant kinds of monoterpenoids, which possessed the highest contents of several volatiles at stage 2. Contrarily, HP were rich in sesquiterpenoids, whose volatile contents were the highest at stage 1.

### 2.3. Correlation Analysis

Based on the screened TFs, Pearson correlation was performed among TFs and detected volatiles (Table S3). Three TFs belonged to the upregulation group while others were classified in downregulation group. The nucleotide sequences of specific genes were distinguished on NCBI via BLAST and the annotations of those genes in relative species were described in the present study. Two TFs separately from C2H2 and HB families showed negative correlations with cosmene, meanwhile, *Cg5g022560* (*MYB108-like* in *Citrus sinensis*) exhibited a positive correlation. *Cg6g016260* (*HSFA-3* in *Citrus clementina*) was negatively related to five terpenoids and HDA, methyl ester. Three monoterpenoids, including trans-beta-ocimene, beta-ocimene and cosmene performed high correlation values with *Cg3g023200* (*bZIP11* in *Citrus sinensis*) and *CgUng002160* (*CYCLOIDEA-like* in *Citrus sinensis*). *Cg7g004890* (*ZAT9-like* in *Citrus sinensis*) was lonely positively correlated to linalool while *Cg9g020350* (*HSFB-3-like* in *Citrus sinensis*) and *Cg7g016740* (*WRKY 75* in *Citrus sinensis*) correlated with both linalool and caryophyllene oxide. Predominantly, *Cg5g019440* (*GRF2* in *Citrus clementina*) showed positive correlation value with several sesquiterpenoids. Besides, close relationships were detected in *Cg7g010390* (*WRKY75* in *Citrus japonica*) with trans-limonene oxide, caryophyllene and caryophyllene oxide.

**Figure 5:**
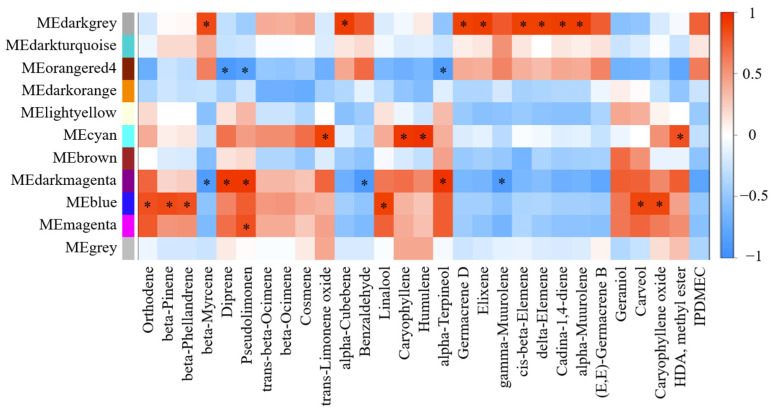
Gene-traits correlation heatmap of WGCNA results. HDA: Hexadecanoic acid; IPDMEC: (1R,7S,E)-7-Isopropyl-4,10-dimethylenecyclodec-5-enol.

Apart from the above findings, weighted gene co-expression network analysis (WGCNA) was performed on transcriptomic results and all the detected volatiles. As shown in Figure 5, monoterpenoids orthodene, beta-pinene, beta-phellandrene, linalool and carveol were correlated to Blue Module. Darkgrey Module showed correlations with sesquiterpenes, including alpha-cubebene, germacrene D, elemenes and alpha-muurolene while Orangered4 Module was negatively correlated to monoterpenoids, including diprene, pseudolimonen and alpha-terpineol. Diversely, pseudolimonen was positively correlated to Magenta and Darkmagenta Modules, meanwhile, diprene and alpha-terpineol were correlated to Darkmagenta Module, which also exhibited negative correlation with beta-myrcene and gamma-muurolene. Trans-limonene oxide, caryophyllene and humulene performed similarly to Cyan Module. The uniquely detected benzaldehyde also showed negative correlation with Darkmagenta Module. Besides, HDA, methyl ester held positive correlation value with Cyan Module. Then, TFs enrichments were conducted in each module for discussion (Table S4).

## 3. Discussion

### 3.1. New Insight into the Volatiles Composition of Young Pomelo Fruits

With the increasing studies on pomelos, the abundant volatiles component has been widely reported, including the volatiles composition in versatile pomelo varieties [12], the representative volatiles in pomelo tissues that provided unique flavor and aroma [13], as well as the volatiles variation during pomelo fruits ripening [1]. However, young pomelo fruits, playing distinctive roles in extractions of bioactive compounds, were limited in volatiles investigation. Our study focused on the variation of volatiles during young pomelo fruits development with a total 29 components detected. Diprene, regarded as the principal component with enormous content in young pomelo fruits, was a natural compound emitted from *Zingiber montanum* and *Zingiber officinale*. Despite the rare reports concerning its aroma, the rich content might provide the pungent, ginger-like special smell in young pomelos.

Consistent with our results in GP, beta-ocimene has been reported as a main volatile compound in the essential oils of *Citrus sinensis*, *Citrus grandis* and *Citrus aurantifolia* leaves while caryophyllene was detected as the main sesquiterpene in the essential oils of *Citrus grandis* and *Citrus aurantifolia* leaves [14]. beta-Ocimene is a ubiquitous volatile in floral scents, whose roles in flowers and other plant tissues were well-established [15], the high content at the initial stages in GP young pomelo fruits development could provide feedstocks for extraction of floral fragrance. Besides, caryophyllene presents a woody spicy odor and plays a role as a potential pharmaceutical agent with its potent anti-inflammatory activity [16]. It has been evaluated as a safe food additive by sixty-third meeting of the Joint FAO/WHO Expert Committee on Food Additives (JEFCA) [17]. Hence, GP could also serve as raw materials for usages in medical and food industries.

beta-Myrcene is an unsubstituted monoterpene with a pleasant smell which generates spontaneously in the essential oils of plants including citrus fruits and citrus juices [18]. As reported in *Citrus depressa*, rather than in juice, the abundant volatile compounds were mainly accumulated in oil glands of peel with significant high concentrations [19]. In the early development periods of pomelos, peels comprised the majority portions of the whole fruits. Hence similar to HP in our results, He et al. investigated beta-myrcene as one of the principal components in pomelo peel essential oil [20]. The HP young pomelo fruits provided an alternative source of beta-myrcene for food additives that potentially exerted health benefits [18]. In addition to beta-myrcene, germacrene D was also reported as a major volatile in *Citrus maxima* peel [21], which was abundant in HP young pomelo fruits in our results. It served as the major flavor contributor in pomelo peels thus was an excellent material of essence. In general, the two varieties of young pomelos fruits, GP and HP, expressed diverse flavor and odor owing to their different principal volatile components, which could be extracted for the versatile usages in different walks of life.

### 3.2. The Variations of Volatiles during Fruit Development

Hundreds of volatile compounds have been reported in citrus fruits, among which monoterpenes and sesquiterpenes are responsible for characteristic aroma [22]. In the case of our results, majority of volatiles were decreased during young pomelo fruits development. Caryophyllene and humulene exhibited negatively to time patterns both in GP and HP. In grape berries, alpha-humulene was one of the terpenes that characterized the early berries development, but benzene derivatives were predominant in later stages [23]. Mehta et al. identified the reductions of relative abundances of terpenes, including alpha-humulene and caryophyllene, when passing from the unripe to the half-ripe maturation stages in jambolana fruit [24]. Besides, unripe fruits possessed more volatiles than the fruits in half-ripe and ripe stages and the decreases of volatile compounds were detected during maturation [24]. These findings were consistent with our results, which also explained the mainstream changes of volatiles during young pomelo fruits development. In addition, it was reported in *Citrus sinensis* that cuticle thickness continuously decreased during the fruit maturation process [25]. Taking the predominantly accumulated volatiles in oil glands of peel into consideration [19], during the development of young pomelos, the cuticle kept growing and relatively resulted in the reducing proportion of peel, hence terpenoids were detected with declinations.

Dramatically, significant accumulations of plenty of volatiles were detected in GP at stage 2. On the one side, apart from the easily released free volatiles, bound volatile components conjugated with glycosides, could be liberated during fruit maturation and released free volatiles to achieve the enhancement of aroma quality [22]. On the other side, the oil glands of peel were responsible for the accumulation of abundant volatile compounds [19]. In *Rubus corchorifolius* fruit, both free and bound terpenoids declined during ripening [26]. Our results concerning GP young pomelos seemed to identify the best period (stage 2) for collecting free volatiles: with the development from stage 1 to stage 2, bound volatiles probably liberated and consequently enhanced free volatiles, meanwhile, oil glands in peels became rich for preserving volatiles. Besides, terpenoids in HP were witnessed with sharp declinations between stage 1 and stage 2. Hence indicated that the stage 1 of HP fruits perhaps corresponded to stage 2 of GP fruits, and HP fruits in stage 1 could be the preferable materials for essential oil extraction.

### 3.3. The Potential Regulatory Roles of TFs on Volatiles Accumulation

The prominently identified volatiles in young pomelo fruits were monoterpenoids as well as sesquiterpenoids, whose biosynthesis pathways were poorly understood but mainly epitomized under the catalyzation of different terpene synthases on linear prenyl compounds, geranyl diphosphate and farnesyl diphosphate, respectively [27]. Transcriptomic analysis was widely operated on citrus to excavate key TFs on volatile terpenoids metabolism involving four TF families (AP2/ERF, bHLH, WRKY, and bZIP) [27], but the investigations on pomelo fruits are relatively limited, which mainly focus on carotenoids accumulation at present [10].

The screened 29 TFs that were consistently up- and down-regulated during young pomelo fruits development were further analyzed with volatiles (Table S3). WRKY75 was widely-studied in *Arabidopsis* with the roles on regulating leaf senescence [28]. In addition, six *WRKY* unigenes were highly correlated with eight deduced *TPS* unigenes in *Amomum villosum* Lour., which indicated the roles of *WRKYs* on terpenoids metabolism [29]. Therefore, the down-regulation of *WRKY75* during pomelo fruits development in our results was reasonable and probably participated in mediating terpenoids delination. As afore studied [30], bZIP family had modulatory functions on terpenoids biosynthesis, as *AabZIP1* could promote the biosynthesis of artemisinin in transgenic *Artemisia annua* plants. Hence the results in the present study promoted to assume a regulatory network of *bZIP11* on monoterpenoids variation during young pomelo fruits development.

In WGCNA results, Blue Module showed close relationships with monoterpenoids, which comprised 33 TFs (Table S4). Among them, *Cg1g019920* (*ERF003-like* in *Citrus sinensis*) might play important roles on monoterpenoids accumulation, supported by the identified possible regulatory role of *ERF003* on carotenoid metabolism during apricot ripening in a former study [31]. Besides, MYC2 was widely studied as a key regulator on terpenoid biosynthesis [32], whose variation pattern was classified into Blue Module (*Cg5g040200*: *MYC2* in *Citrus clementina*) and highly correlated to monoterpenoids accumulation in our study. In *Artemisia annua*, the overexpression of *AaMYC2* enhanced the content of a sesquiterpene lactone, artemisinin, by directly binding to the promoters of synthetic genes via the modulation of jasmonic acid, meanwhile, caused the loss of a competitive pathway product, artemisinic acid [32]. The three volatile components, which highly correlated with Cyan Module, consistently decreased during young pomelo fruits development, which might be straightly mediated by metabolic processes during fruits ripening. *Cg9g000740* (*MYB108* in *Citrus clementina*) was enriched in Cyan Module. In cotton, *MYB108* was reported relative to fiber development, which was up-regulated in cotton line with high fiber strength [33]. During the development of young pomelo fruits, the relative fiber content was correspondingly decreased with the reduced proportion of peel, hence *MYB108* down-regulated. Besides, *Cg6g025130* (*NAC100* in *Citrus clementina*) was putatively involved in regulation networks to contribute in fruit ripening of pomelos (*Citrus grandis Osbeck*) [34]. Hence the components correlated to Cyan Module could be used in discriminating young pomelo fruits at different developing stages. In Darkgrey Module, 31 TFs were identified, including TF families AP2/ERF, bHLH, NAC, WRKY, ZF-HD, etc., which tend to regulate the formation of sesquiterpenoids. Nevertheless, the transcriptomic results together with volatile profiles in young pomelo fruits could enrich the knowledge of the potential interactions and regulatory network. As previously reported, the overexpression of specific genes in terpenoids biosynthesis pathway is effective for the enhancement yield of terpenoids [27]. For example, a group of triterpenoids, ginsenosides, was able to be accumulated by overexpressing the relative genes on biosynthetic pathway [27]. Besides, the overexpression of *Clarkia breweri* linalool synthase gene in *Lavandula latifolia* plants conduced significant increases of linalool, which is the appreciated compound in perfume and cosmetic industries [35]. Therefore, the manipulation of relative genes for volatile biosynthesis in young pomelo fruits could be a novel strategy to accumulate large amounts of useful aromas for industrial use, which should be processed with further investigation.

## 4. Materials and Methods

### 4.1. Sample Collection

Two genotypes of young pomelos Golden Pomelo (GP) and Honey Pomelo (HP) studied in the present work were grown in Meizhou Honey Pomelo National Industrial Park (Meizhou, China; 24°19′ N, 116°07′ E) with systematical management. The growing condition could be referred to the published work [11]. Each variety of young pomelos were collected at four developmental stages (Stage 1 to 4), which were divided by pomelos diameters around 2, 4, 6, and 8 cm (corresponding days after pollen: 30, 40, 47 and 54), and were from three pomelo trees with similar growth patterns. At least 10 fruits were taken from each tree at each stage for analysis. The fresh young pomelos were washed, frozen in liquid nitrogen and stored at −80 °C until use. GP and HP separately collected at four stages were labelled as GP1 to 4 (Golden Pomelo collected at stage 1 to 4) as well as HP1 to 4 (Honey Pomelo collected at stage 1 to 4).

### 4.2. Transcriptomic Analyses

Samples were sent to Biomarker (Beijing, China) in dry ice for transcriptomic analysis via RNA sequencing method. Oligo (dT) beads were used to enrich mRNA from the qualified RNA. Then, mRNA was randomly broken into fragments and reverse-transcribed into cDNA. After purifying, repairing and screening, cDNA was enriched by PCR and corpus were constructed. Sequencing was operated on Illumina platform. Totally, 150.61Gb clean data were created and aligned to *Citrus grandis* (L.) Osbeck.cv. ‘Wanbaiyou’ v1.0 (available at http://citrus.hzau.edu.cn, accessed on 28 June 2021) via HISAT2 system. The unique mapped reads were around 88 to 93%. Besides, 1697 new genes were identified and annotated by DIAMOND and HMMER software.

### 4.3. Volatiles Determination

The determination of volatiles was conducted on GC-MS/MS system according to the previous study with modification [36]. Pomelo fruits were powdered by liquid nitrogen and weighed 1 g for analyzation. Samples were preheated at 65 °C for 15 min. Then, volatiles were absorbed by extraction fiber for 45 min and desorbed for 4 min. Experiments were operated on gas chromatography system (HS-SPME-GC, Aglient Technologies 7890B, Palo Alto, CA, USA) combined with a triple quadrupole-MS (TQ-MS, 7000C GC/MS Triple Quad, Aglient Technologies, Palo Alto, CA, USA). The initial condition of capillary column (HP-INNOWax, 30 m × 0.25 mm, 0.25 μm, Aglient Technologies, Palo Alto, CA, USA) was 50 °C for 3 min, and firstly increased to 140 °C at a rate of 5 °C/min, after keeping for 3 min, temperature was then increased to 250 °C at a rate of 5 °C/min and maintained at 260 °C for 3 min. The injection port temperature was set as 250 °C. The splitter was flush with helium at 1 mL/min. L-2-octanol was added to samples as an internal standard for semi-quantification and mass spectrogram of each compound was compared to NIST 2020 library (Agilent Technologies G1033A, Palo Alto, CA, USA) for qualification. Results were expressed as ng g^−1^ fresh weight (FW) (*n* = 3).

### 4.4. Weighted Gene Co-Expression Network Analysis (WGCNA)

WGCNA was conducted on platform BMKCloud (available at www.biocloud.net, accessed on 20 March 2022). Genes from 24 samples were screened as FPKM ≥ 1. The minimum module size was 30 and the minimum height for merging modules was 0.25, separately for the high reliability of the results.

### 4.5. Statistic Analysis

Most of the transcriptomic analyses were operated on BMKCloud (available at www.biocloud.net, accessed on 20 March 2022), including KEGG enrichment and transcription factor prediction. Differently expression genes (DEGs) were screened by FC > 2 and FDR value < 0.01 on DESeq2. Time cluster number was set up as 9, and the FPKM values of genes were normalized for analysis. Venn plot was performed on jvenn [37]. Principal components analysis and correlation analysis on volatiles and development stages were accomplished on MetaboAnalyst5.0. Genes were labelled according to transcriptomic results, in which their nucleotide sequences were recorded and were used for BLAST on NCBI for the sake of annotating relative functions. Significant differences were analyzed via Tukey method on IBM SPSS Statistics 25.0 (SPSS Inc., Chicago, IL, USA) (*p* < 0.05). All determinations were measured in triple.

## 5. Conclusions

Summarily, 29 volatiles were identified during young pomelo fruits development, which were mainly classified into monoterpenoids and sesquiterpenoids. Diprene was the principal component, whose concentration was the highest among the detected volatiles. Monoterpenoids were abundant in GP whereas sesquiterpenoids were highly accumulated in HP. During fruits development, majority volatiles were gradually decreased. GP2 and HP1 enjoyed the most abundant volatiles in diverse varieties, which were preferable materials for essential oil extraction in order to meet the needs of all works of life. Besides, 12 and 17 TFs were consistently up- and down-regulated in both GP and HP varieties during fruits development, thus probably played roles in regulating young pomelos metabolism. WGCNA analysis figured out two modules that distinctly correlated to monoterpenoids and sesquiterpenoids accumulation. The potential functions of *ERF003* and *MYC2* on monoterpenoids metabolism were predicted in our study. Therefore, the present study, by reporting the volatile profiles during pomelo fruits development, provided guidance for young pomelo fruits shedding as well as additional feedstocks for essential oils, meanwhile, increased the knowledge of potential regulatory mechanism of TFs on volatiles accumulation, which could be exerted for specific volatile accumulation with further investigation.

## Data Availability

Not applicable.

## Supplementary Materials

The following supporting information can be downloaded at: https://www.mdpi.com/article/10.3390/ijms23105665/s1.
