# Supplementary material for: Volatiles Accumulation during Young Pomelo (Citrus maxima (Burm.) Merr.) Fruits Development
Source: Int J Mol Sci. 2022 May 18;23(10):5665. doi: 10.3390/ijms23105665 (PMC9144960; doi:10.3390/ijms23105665)
Supplement: Supplementary file 1 [file ijms-23-05665-s001.zip › Table S2.pdf]

Table S2. Volatiles in young pomelo fruits (μg g^{-1}, mean ± SD, n = 3)

No.	Name	RT (min)	GP1	GP2	GP3	GP4	HP1	HP2	HP3	HP4
1	Orthodene	4.83	9.982±0.448c	23.33±1.11a	13.82±0.29b	6.205±0.689de	6.924±0.622d	4.934±0.251e	2.778±0.134f	1.872±0.295f
2	beta-Pinene	6.7	5.396±0.600e	209.7±2.3a	29.12±0.95c	18.29±2.47d	66.17±6.03b	59.29±4.00b	28.57±1.92c	18.87±2.61d
3	beta-Phellandrene	7.09	3.403±0.198f	37.60±0.11a	7.934±0.145d	4.107±0.435ef	13.77±1.30b	11.52±0.74c	5.662±0.319e	3.735±0.504f
4	beta-Myrcene	8.27	57.74±3.83d	64.03±3.37d	65.91±3.11d	29.64±2.66d	699.7±62.7a	521.3±37.5b	330.6±17.9c	258.8±34.3c
5	Diprene	9.14	3218±242ab	3497±111a	3066±287b	1828±126c	1118±89d	580.4±34.1e	481.8±28.3e	357.9±22.2e
6	Pseudolimonen	9.36	8.159±0.539c	14.65±0.90a	10.87±0.29b	5.079±0.575d	ND	ND	ND	ND
7	trans-beta-Ocimene	10.21	8.031±0.581c	13.68±0.51a	6.683±0.318d	1.161±0.109f	11.43±0.91b	3.853±0.327e	1.412±0.100f	0.516±0.079f
8	beta-Ocimene	10.66	353.7±22.1c	595.0±16.1a	299.6±11.9d	56.12±3.43f	489.6±40.2b	174.0±18.5e	67.29±3.76f	25.78±1.64f
9	Cosmene	15.84	3.332±0.392b	4.188±0.073a	1.657±0.134c	ND	3.625±0.504ab	1.372±0.165c	0.540±0.062d	0.263±0.030d
10	trans-Limonene oxide	16.06	3.982±0.367a	2.426±0.323b	1.629±0.131c	ND	ND	ND	ND	ND
11	alpha-Cubebene	15.93	ND	ND	ND	ND	3.871±0.375a	1.276±0.173b	0.759±0.054bc	0.629±0.005c
12	Benzaldehyde	17.88	ND	ND	ND	ND	1.320±0.158a	0.651±0.261b	0.643±0.018b	0.524±0.063b
13	Linalool	18.43	27.24±2.40b	81.57±0.69a	31.52±2.61b	12.88±0.85d	19.53±2.52c	8.936±0.347de	5.149±0.244ef	3.688±0.395f
14	Caryophyllene	19.38	464.9±37.2a	309.1±7.6b	182.6±18.9c	35.51±0.92e	80.22±4.79d	32.74±1.97e	23.97±2.52e	20.30±2.02e
15	Humulene	21.07	42.50±3.71a	27.72±0.61b	16.37±1.72c	3.209±0.059e	15.92±0.41c	6.019±0.298e	4.121±0.478e	4.204±0.372e
16	alpha-Terpineol	21.91	2.715±0.109c	5.308±0.178a	3.884±0.494b	3.410±0.131bc	ND	ND	ND	ND
17	Germacrene D	21.99	22.17±1.80d	24.70±1.96d	96.34±10.79c	3.492±0.352d	633.7±38.1a	207.4±24.4b	135.6±15.4c	121.7±15.5c
18	Elixene	22.54	ND	ND	ND	ND	38.31±0.32a	12.03±0.59b	6.080±0.690c	5.502±0.714c
19	gamma-Muurolene	23.1	ND	ND	ND	ND	13.39±0.48a	12.42±0.01a	3.959±0.275c	6.593±0.890b
20	cis-beta-Elemene	23.18	2.965±0.206c	2.776±0.29c	3.333±0.245c	ND	11.98±1.13a	5.536±0.320b	3.536±0.314c	4.073±0.538c

21	delta-EIemene	23.45	ND	ND	ND	ND	4.611±0.258a	0.756±0.010b	0.511±0.017bc	0.416±0.021c
22	Cadina-1,4-diene	23.61	ND	ND	ND	ND	1.437±0.119a	0.462±0.040b	0.284±0.008c	0.231±0.010c
23	alpha-Muurolene	23.84	ND	ND	ND	ND	3.627±0.205a	1.050±0.115b	0.607±0.071c	0.587±0.082c
24	(E,E)-Germacrene B	24.62	ND	ND	ND	ND	12.93±0.92a	5.548±0.321c	1.639±0.156d	7.254±0.239b
25	Geraniol	25.22	ND	3.456±0.036b	3.333±0.245b	3.985±0.042a	ND	ND	ND	ND
26	Carveol	25.63	ND	3.258±0.089a	1.754±0.187c	2.133±0.017b	ND	ND	ND	ND
27	Caryophyllene oxide	27.84	6.158±0.121b	14.17±1.50a	2.099±0.511c	0.974±0.016c	1.282±0.142c	1.098±0.071c	1.379±0.231c	0.997±0.088c
28	HA, methyl ester	32.25	8.695±1.158a	7.566±0.445a	6.179±0.258b	2.417±0.370d	3.939±0.089c	1.585±0.084de	0.826±0.133e	1.701±0.255de
29	IPDMEC	34.91	ND	ND	ND	ND	2.362±0.033a	1.013±0.204c	1.745±0.081b	0.296±0.005d

* RT: Retention time; HDA: Hexadecanoic acid; IPDMEC: (1R,7S,E)-7-Isopropyl-4,10-dimethylenecyclodec-5-enol; ND: No detected.

* Different letters in a line stand for significant differences ($p < 0.05$).
