# Supplementary material for: Volatiles Accumulation during Young Pomelo (Citrus maxima (Burm.) Merr.) Fruits Development
Source: Int J Mol Sci. 2022 May 18;23(10):5665. doi: 10.3390/ijms23105665 (PMC9144960; doi:10.3390/ijms23105665)
Supplement: Supplementary file 1 [file ijms-23-05665-s001.zip › Table S3.pdf]

Table S3. Results of Pearson correlation between TFs and volatiles.

Gene ID	Cg2g028970	Cg1g014500	Cg6g016260	Cg3g023200	Cg7g004890	Cg5g019440	Cg9g020350	Cg5g022560	CgUng002160	Cg7g010390	Cg7g016740
TF family	C2H2	HB	HSF	bZIP	C2H2	GRF	HSF	MYB	TCP	WRKY	WRKY
trans-beta-Ocimene	-0.761	-0.744	**-0.823**	**0.921**	0.890	0.564	0.743	0.862	**0.941**	0.670	0.710
beta-Ocimene	-0.760	-0.744	**-0.826**	**0.917**	0.894	0.553	0.746	0.857	**0.938**	0.677	0.715
Cosmene	**-0.859**	**-0.820**	**-0.880**	**0.940**	0.892	0.609	0.741	**0.927**	**0.911**	0.732	0.698
trans-Limonene oxide	-0.478	-0.442	-0.780	0.432	0.774	-0.104	0.651	0.500	0.363	**0.910**	0.727
alpha-Cubebene	-0.454	-0.432	-0.096	0.435	-0.131	**0.904**	-0.243	0.430	0.529	-0.408	-0.365
Linalool	-0.373	-0.365	-0.590	0.642	**0.904**	0.028	**0.902**	0.532	0.585	0.805	**0.917**
Caryophyllene	-0.549	-0.524	**-0.835**	0.536	0.826	0.002	0.703	0.607	0.458	**0.930**	0.762
Humulene	-0.675	-0.637	**-0.895**	0.647	0.837	0.190	0.684	0.727	0.584	0.896	0.721
Elixene	-0.465	-0.446	-0.117	0.452	-0.104	**0.908**	-0.222	0.452	0.555	-0.388	-0.340
cis-beta-Elemene	-0.630	-0.525	-0.236	0.511	0.006	**0.950**	-0.180	0.503	0.642	-0.271	-0.276
Cadina-1,4-diene	-0.453	-0.431	-0.099	0.438	-0.130	**0.903**	-0.242	0.431	0.531	-0.407	-0.363
alpha-Muurolene	-0.453	-0.427	-0.110	0.447	-0.112	**0.902**	-0.227	0.443	0.549	-0.391	-0.342
Caryophyllene oxide	-0.401	-0.364	-0.580	0.623	0.891	-0.011	**0.952**	0.552	0.444	**0.902**	**0.947**
HDA, methyl ester	-0.528	-0.504	**-0.837**	0.580	0.881	0.047	0.716	0.620	0.613	0.882	0.797

* The bold values were discussed in manuscript.

* HDA: Hexadecanoic acid
